# Association analysis of single nucleotide polymorphisms in autophagy related 7 (*ATG7*) gene in patients with coronary artery disease

**DOI:** 10.1097/MD.0000000000029776

**Published:** 2022-06-30

**Authors:** Moomal Sarosh, Syed Muhammad Nurulain, Syed Tahir Abbas Shah, Muhammad Jadoon Khan, Zahid Muneer, Nazia Bibi, Syed Fawad Ali Shah, Sabir Hussain

**Affiliations:** a Department of Biosciences, COMSATS University Islamabad, Islamabad, Pakistan.

**Keywords:** ATG7, autophagy, coronary artery disease, gene, polymorphism

## Abstract

Recent experimental studies sparked the involvement of autophagy-related 7 (ATG7) in the development of atherosclerosis. However, the genetic variants and their association with coronary artery disease (CAD) are still to be unveiled. Therefore, we aimed to design a retrospective case-control study for the analysis of *ATG7* gene polymorphisms and their association with CAD among the subjects originating from Pakistan.

The *ATG7* noncoding polymorphisms (rs1375206; Chr3:11297643 C/G and rs550744886; Chr3:11272004 C/G) were examined in 600 subjects, including 300 individuals diagnosed with CAD. Arginase-1 (ARG1) and nitric oxide metabolites were measured by the colorimetric enzymatic assay. Genotyping of noncoding *ATG7* polymorphisms was accomplished by the polymerase chain reaction–restriction fragment length polymorphism method.

A significant association of *ATG7* (rs1375206 and rs550744886) was observed in individuals exhibiting CAD (*P* < .0001, for each single-nucleotide polymorphism). Moreover, variant allele G at both loci showed high occurrence and significant association with the disease phenotype as compared to the wild-type allele (odds ratio [OR] = 2.03, *P* < .0001 and OR = 2.08, *P* < .001, respectively). Variant genotypes at *ATG7* rs1375206 and rs550744886 showed significant association with high concentrations of ARG1 and low nitric oxide metabolites among the patients (*P* < .0001 for each). A significant difference was noted in the distribution of the haplotype G-G, mapped at Chr3:11297643-11272004 between cases and controls (*P* < .0001).

The study concludes that *ATG7* polymorphisms are among the risk factors for CAD in the subjects from Pakistan. The study thus highlights the novel risk factors for high incidents of the disease and reported for the first time to the best of our knowledge.

## 1. Introduction

Coronary artery disease (CAD) is a multifactorial ailment, which has the highest mortality and morbidity rates globally. A number of traditional or conventional risk factors like cholesterol, low-density lipoprotein (LDL), hypertension, triglycerides (TGs), smoking, and obesity contribute to CAD.^[1]^ Currently, available treatment strategies targeting traditional risk factors could not limit the ever-rising number of CAD cases in different populations. Now the scientific community is focusing their struggles to identify novel CAD risk factors to broaden the therapeutic targets and to deal with the high incidence of CAD cases. Several proteins from the metabolic pathway of autophagy have been identified and are being used for the best risk assessment of CAD.^[2]^ One such protein is autophagy-related 7 (ATG7), relatively novel and has been implicated in the pathophysiology of cardiovascular diseases.^[3]^ ATG7 (OMIM: 608760) is an E1-like activating enzyme, which acts as a critical mediator in the biogenesis of autophagosomes.^[4]^ ATG7 is dual in nature: first, it acts as a ligase to conjugate ATG12 with ATG5, playing a central role in the generation of the functional autophagosome.^[5]^ Subsequently, ATG7 acts on immature microtubule-associated protein 1 light chain 3 to develop autophagosomal membrane protein by inserting phosphatidylethanolamine.^[4]^

ATG7’s significance in increasing inflammation and cardiovascular disease events has been demonstrated in several animal studies.^[6]^ The systemic deletion of *Atg7* produces harm to normal autophagy, indicating that ATG7 plays a significant role in atherosclerosis.^[7]^ Defective autophagy, especially specific deletion of *Atg7* from smooth muscle cells, promotes unstable plaque phenotype in apolipoprotein E–deficient mice.^[8]^ Recently, Zhang et al^[3]^ reported that *ATG7* variants might reduce the transcriptional activity of the *ATG7* gene, resulting in the development of acute myocardial infarction in patients. However, single-nucleotide polymorphic sites in *ATG7* and their association with CAD have not been reported. Therefore, we aimed to study the association between genetic variants in *ATG7* and CAD among the patients. It has been proposed for the first time in the present study that variant genotypes and alleles at *ATG7* rs1375206; Chr3:11297643 and rs550744886; Chr3:11272004 confer the risk of CAD. Moreover, the variant genotype at both loci showed a tight link with high arginase-1 (ARG1) and low nitric oxide metabolites in CAD patients.

## 2. Materials and methods

### 2.1. Study type

Retrospective case-control association study.

### 2.2. Study duration

January 2019 to April 2021.

### 2.3. Subjects

The present research presented a genetic analysis of *ATG7* in the subjects affected by CAD as well as in healthy volunteers. The study protocol conformed to the principles of the Helsinki Declaration, 2002 followed by ethical approval from the Ethics Review Board of COMSATS University Islamabad. All the patients and controls signed an informed consent form during the sample collection. Three hundred CAD patients (258 males and 42 females) and 300 healthy controls (248 males and 58 females) were enrolled. Subjects in the CAD patient group who had any other systemic cardiovascular disease except CAD were not included in the study. Patients were evaluated by clinical and physical examination. Similarly, healthy volunteers were enrolled from the same localities after evaluation of a normal electrocardiogram/echocardiogram/exercise tolerance test and a history of any cardiovascular disease. CAD was defined on the basis of angiographic criteria developed by Ledru et al.^[9]^ Moreover, age and gender matching were followed between patients and controls.

### 2.4. Sample processing

Standard techniques were adopted for the collection of venous blood samples/serum from the participants.^[10]^ Serum was stored at −80°C for the analysis of biochemical and clinical markers. The nonenzymatic salting out approach was used to extract deoxyribonucleic acid (DNA) from whole blood for single-nucleotide polymorphism (SNP) analysis.^[11]^

### 2.5. Biochemical analyses

The lipid profile (total cholesterol [TC], TG, LDL, and high-density lipoprotein [HDL]) of each subject was determined by using AMP colorimetric assay kits (AMP Diagnostics, Austria). Measurements were carried out in the SPECORD 50 PLUS chemistry analyzer (Analytik Jena, Germany). Serum levels of nitrite, nitrate, and ARG1 were determined by enzyme-linked immunosorbent assay technique as described earlier.^[10]^

### 2.6. Genotyping of ATG7 SNPs

Genotyping of *ATG7* rs1375206; Chr3:11297643 was carried out by polymerase chain reaction (PCR) using forward primer 5′-TGAGAGCAGTGCTTTAACACC-3′ and reverse primer 5′-TGAAATAATAAAATATATATCA-3′. Similarly, rs550744886; Chr3:11272004 was amplified by forward primer 5′-AGCTGTGAGAATGATGGCTGG-3′ and reverse primer 5′-GCCATCAGGGTGGCAGGTG -3′. The 50 µL of PCR reaction contained 3 µL of genomic DNA, 5 µL of 10× PCR buffer (0.2 M of (NH4)_2_SO_4_, 0.8 M Tris–hydrochloric acid [HCl] (pH 8.8), and 0.2% w/v of Tween-20), 4 µL of 25 mM MgCl_2_ (Solis BioDyne, Tartu, Estonia), 0.5 µL (5 U/µL) of Taq DNA polymerase (Solis BioDyne, Tartu, Estonia), 1 µL of 20 mM deoxynucleotide triphosphates (Solis BioDyne, Tartu, Estonia), 2.5 µL of each forward and reverse primer, and 31.5 µL of PCR grade water. Each reaction was performed in ProFlex PCR System (Applied Biosystems). PCR reactions were carried out with the thermal cycling conditions including an initial denaturation of DNA at 95°C for 10 minutes, followed by amplification for 35 cycles at 95°C for 30 seconds, 60°C for 1 minute, and 72°C for 2 minutes. The final extension step was run at 72°C for 10 minutes. Electrophoresis on a 2% agarose gel was used to evaluate the PCR amplified products. The genotype pattern of both SNPs in *ATG7* was performed by restriction fragment length polymorphism. Restriction enzymes (Thermo Scientific) Bsp1286I (for rs1375206) and H*ae*III (for rs550744886) were used for the SNP genotyping, respectively. Twenty microliters of the reaction mixture was prepared for both enzymes using 12 µL of PCR products, 2 µL of G (10×) buffer (10 mM Tris–HCl, 10 mM MgCl_2_, 50 mM NaCl, and 0.1 mg/mL bovine serum albumin), and 0.5 µL of each enzyme in 5.5 µL of PCR grade water. The reaction tubes were incubated at 37°C for 18 hours, and the products were visualized on 3% agarose gel using electrophoresis.

### 2.7. Statistical calculations

Statistical analyses were performed by GraphPad InStat 3 (GraphPad Software Inc, San Diego, CA). Mean, standard deviation, frequencies, and percentage were quality metrics of data. For comparing biochemical and demographic parameters, the χ^2^ test and independent samples *t* test were applied. Frequency percentage of age, smoking, and gender was obtained by MedCalc (MedCalc Software Ltd, Belgium). Association was studied by comparing genotype and allele frequencies between patients and controls by χ^2^ test using 2 × 3 contingency table and Fisher exact test using 2 × 2 contingency table, respectively. *P* < .05 was set as significant for the association throughout the study.

## 3. Results

### 3.1. Demographic and biochemical characteristics of subjects

An independent cohort of 600 subjects including 300 CAD patients with the mean age of 44.16 ± 9.3 years and 300 healthy controls with the mean age of 44.20 ± 7.3 years were recruited in this study. There were 86% males and 14% females in the patient group, while 82.7% males and 17.3% females were enrolled in the healthy control group. There was no significant difference in the distribution of age and gender between the patients and controls (*P* > .05, respectively; Table 1). Nonsignificant distribution was found for tobacco smoking and body mass index (BMI) between the CAD patients and controls (*P* > .05 for each; Table 1). Systolic blood pressure and diastolic blood pressure were significantly high among the CAD patients as compared with controls (*P* < .0001, respectively; Table 1). Among the lipid biomarkers, TC, TG, and LDL were significantly higher in CAD patients than in controls (*P* < .0001 for each; Table 1). Good cholesterol (HDL) was significantly lowered (34.84 ± 8.1 mg/dL) in CAD patients compared with 38.61 ± 9.5 mg/dL in healthy volunteers (*P* < .0001; Table 1). Moreover, serum ARG1 levels were significantly associated with CAD at 76.37 ± 29.4 ng/mL among the patients as compared with 45.49 ± 12.3 ng/mL in healthy controls (*P* < .0001; Table 1). Serum nitrate (13.77 ± 8.0 µM) and nitrite (0.40 ± 0.19 µM) were significantly lower in CAD patients than (nitrite = 0.65 ± 0.25 µM, nitrate = 29.43 µM) in healthy controls (*P* < .0001 for each; Table 1).

**Table 1:** Baseline and clinical parameters in CAD patients and controls.

Variables	CAD patients (n = 300)	Controls (n = 300)	*P* value
Age (yr)	44.16 ± 9.3	44.20 ± 7.3	.9497*
Gender (male/female)	258 (86%)/42 (14%)	248 (82.7%)/52 (17.3%)	.3121†
BMI (kg/m^2^)	25.54 ± 2.3	25.73 ± 2.2	.3068*
Physical activity (yes/no)	120 (40%)/180 (60%)	212 (70.7%)/88 (29.3%)	<.0001†
Smoking (yes/no)	65 (21.7%)/235 (78.3%)	59 (19.7%)/241 (80.3%)	.6142†
SBP (mm Hg)	127.72 ± 13.6	121.24 ± 4.4	<.0001*
DBP (mm Hg)	86.67 ± 8.1	79.73 ± 7.3	<.0001*
Total cholesterol (mg/dL)	190.10 ± 21.3	178.42 ± 18.8	<.0001*
Triglycerides (mg/dL)	100.09 ± 36.3	84.93 ± 14.9	<.0001*
Low-density lipoprotein (mg/dL)	85.34 ± 18.4	81.32 ± 12.8	.0020*
High-density lipoprotein (mg/dL)	34.84 ± 8.1	38.61 ± 9.5	<.0001*
Nitrite levels (µM)	0.40 ± 0.19	0.65 ± 0.25	<.0001*
Nitrate levels (µM)	13.77 ± 8.0	29.43 ± 5.0	<.0001*
Arginase-1 levels (ng/mL)	76.37 ± 29.4	45.49 ± 12.3	<.0001*

Values are given as mean ± SD and percentage.

BMI = body mass index, CAD = coronary artery disease, DBP = diastolic blood pressure, n = number of subjects, SBP = systolic blood pressure, SD = standard deviation.

* *P* values were calculated by using the independent samples *t* test.

† *P* values were calculated by χ^2^ test.

### 3.2. *ATG7* rs1375206 C/G and rs550744886 C/G polymorphism

Genotype frequencies of rs1375206 and rs550744886 in the *ATG7* gene are shown in Table 2. The distribution of the CC, CG, and GG genotypes of *ATG7* rs1375206 and CC, CG, and GG genotypes of *ATG7* rs550744886 among the controls was in Hardy-Weinberg equilibrium (χ^2^ = 2.2, *P* = .1380; χ^2^ = 1.4, *P* = .2367, respectively). The carriers with rs1375206 GG genotype were more prevalent at 18.7% in CAD patients than 8.3% in controls. Heterozygous CG carriers were 45.3% in the CAD patients’ group and 35% in controls. These genotypic variants were found to be significantly associated with CAD in patients compared with controls (χ^2^ = 29.68, *P* < .0001; Table 2).

**Table 2:** Genotype and allele dissemination of *ATG7* rs1375206 C/G and rs550744886 C/G polymorphisms in coronary artery disease patients and controls.

	Patients (n = 300)	Controls (n = 300)	χ^2^ (*P* value)
*ATG7* rs1375206 C/G polymorphism			
CC genotype	108 (36%)	170 (56.7%)	χ^2^ = 29.68,
CG genotype	136 (45.3%)	105 (35%)	*P* < .0001*
GG genotype	56 (18.7%)	25 (8.3%)	
C allele	352 (58.7%)	445 (74.2%)	OR = 2.03,
G allele	248 (41.3%)	155 (25.8%)	95% CI = 1.58–2.58,
			*P* < .0001† (G vs C)
*ATG7* rs550744886 C/G polymorphism			
CC genotype	117 (39%)	180 (60%)	χ^2^ = 30.89,
CG genotype	133 (44.3%)	100 (33.3%)	*P* < .0001*
GG genotype	50 (16.7%)	20 (6.7%)	
C allele	367 (61.2%)	460 (76.7%)	OR = 2.08,
G allele	233 (38.8%)	140 (23.3%)	95% CI = 1.62–2.68,
			*P* < .0001† (G vs C)

Values are given as the number of alleles and genotypes with percentages in parenthesis.

CI = confidence interval, OR = odds ratio.

* *P* values were calculated by χ^2^ test.

† *P* values were calculated by Fisher exact test.

The rs1375206; Chr3:11297643-G variant allele is significantly associated with increased risk of CAD in subjects (odds ratio [OR] = 2.03, 95% CI = 1.58–2.58, *P* < .0001, G vs C; Table 2). Similarly, minor allele G at *ATG7* rs550744886; Chr3:11272004-G showed a significant association with CAD in subjects compared with that in controls (OR = 2.08, 95% CI, 1.62–2.68, *P* < .0001, G vs C; Table 2).

### 3.3. Genotype-wise distribution of biochemical and clinical variables in CAD patients

Patients with CAD showed a varying concentration of biochemical and clinical markers regarding *ATG7* rs1375206 C/G and rs550744886 C/G polymorphism (Table 3). *ATG7* rs1375206 and rs550744886 variant genotypes showed a significant association with TC, TG, LDL, and HDL (*P* < .0001 for each; Table 3). Carriers of the rs1375206 CG + GG and rs550744886 CG + GG showed maximal levels of serum ARG1 in CAD patients than in carriers with CC genotype (*P* < .0001, respectively; Table 3). Moreover, minimal levels of serum nitrite and nitrate in patients were found in the carrier of rs1375206 CG + GG and rs550744886 CG + GG genotypes than in carriers of CC genotypes (*P* < .0001, respectively; Table 3).

**Table 3:** Relationship of *ATG7* rs1375206 C/G and rs550744886 C/G polymorphism with clinical parameters in coronary artery disease patients.

Parameters	*ATG7* rs1375206 C/G SNP			*ATG7* rs550744886 C/G SNP		
	CC	CG + GG	*P* value	CC	CG + GG	*P* value
TC (mg/dL)	177.03 ± 17.0	197.4 ± 20.0	<.0001	179.1 ± 19.2	197.0 ± 19.6	<.0001
TG (mg/dL)	81.2 ± 19.1	110.7 ± 39.3	<.0001	85.4 ± 25.6	109.4 ± 39.0	<.0001
LDL (mg/dL)	72.9 ± 15.5	92.3 ± 16.1	<.0001	74.6 ± 16.8	92.1.3 ± 15.9	<.0001
HDL (mg/dL)	42.8 ± 7.5	30.3 ± 4.1	<.0001	41.4 ± 8.5	30.6 ± 4.0	<.0001
ARG1 levels (ng/mL)	41.0 ± 14.1	96.2 ± 11.8	<.0001	49.5 ± 23.5	93.5 ± 17.5	<.0001
Nitrite (µM)	0.61 ± 0.17	0.29 ± 0.06	<.0001	0.56 ± 0.19	0.31 ± 0.10	<.0001
Nitrate (µM)	21.50 ± 6.8	9.4 ± 4.7	<.0001	19.2 ± 8.1	10.2 ± 5.6	<.0001

Values are given as the mean ± SD.

*P* values were calculated using the independent samples *t* test.

ARG1 = arginase-1, HDL = high-density lipoprotein, LDL = low-density lipoprotein, SD = standard deviation, SNP = single-nucleotide polymorphism, TC = total cholesterol, TG = triglyceride.

### 3.4. ATG7 rs375206/rs550744886 haplotype

The haplotype frequencies of *ATG7* rs375206 C/G and rs550744886 C/G polymorphisms in patients and controls are shown in Table 4. The haplotype C-C was considered as a reference for the entire analysis. Haplotype C-G was equally distributed among the patients and controls (*P* > .05; Table 4), while haplotype G-C was slightly more prevalent in controls than in patients (*P* = .04; Table 4). Haplotype G-G showed a significant association with CAD in patients compared with healthy individuals (*P* < .0001; Table 4).

**Table 4:** Haplotype distribution of *ATG7* rs1375206 C/G and rs550744886 C/G polymorphisms in coronary artery disease patients and control subjects.

Haplotypes	Patients (n = 300)	Controls (n = 300)	χ^2^	OR (95% CI)	*P value
C-C	316 (52.7%)	386 (64.3%)	Reference		
C-G	50 (8.3%)	62 (10.4%)	0.0005	0.98 (0.66–1.47)	1.00
G-C	35 (5.8%)	69 (11.5%)	4.3	0.62 (0.40–0.95)	.04
G-G	199 (33.2%)	83 (13.8%)	51.6	2.9 (2.17–3.93)	<.0001

CI = confidence interval, n = number of subjects, OR = odds ratio.

* *P* values were calculated by χ^2^ test (Yates corrected).

### 3.5. Logistic regression analysis of CAD with clinical variables

Logistic regression for the association between CAD and age, gender, BMI, serum ARG1, nitrate, nitrite, *ATG7* rs375206 C/G, and *ATG7* rs550744886 C/G polymorphism are mentioned in Table 5. Serum levels of ARG1 (*P* < .0001), nitrate (*P* < .0001), nitrite (*P* < .0025), *ATG7* rs375206 C/G (*P* < .0001), and *ATG7* rs550744886 C/G (*P* < .0001) showed a significant association with CAD. However, age, gender, and BMI were not associated with CAD (*P* > .05 for each, respectively).

**Table 5:** Logistic regression analysis (binary model) in all subjects when coronary artery disease was used as a response variable and age, gender, BMI, arginase-1, nitrate, nitrite, and the 2 single-nucleotide polymorphisms of *ATG7* were taken as independent variables.

Variable	Coefficient	95% CI	Odds ratio	*P* value
Constant	−2.59382	–	–	.115
Age	−0.0047176	0.96–1.02	0.995	.7339
Gender	0.25310	0.82–2.00	1.288	.2621
BMI	0.036857	0.96–1.11	1.03	.3057
Serum arginase-1 levels	−0.042135	0.94–0.97	0.599	<.0001
Serum nitrate	0.054615	1.02–1.07	1.05	<.0001
Serum nitrite	1.88504	1.93–22.38	6.58	.0025
*ATG7* rs1375206 C/G polymorphism	−0.084363	0.30–0.59	0.430	<.0001
*ATG7* rs550744886 C/G polymorphism	0.85273	0.31–0.60	0.426	<.0001

BMI = body mass index, CI = confidence interval.

## 4. Discussion

CAD is a multifactorial disease thought to be caused by a combination of genetics and environmental risk factors. It is frequently linked to traditional risk factors such as hyperlipidemia and inflammation. Genome-wide association studies have reported an association of a large number of SNPs in genes regulating inflammation, redox potential, and lipid metabolism with cardiovascular disease patients. However, the collective genetic contribution in the pathogenesis of CAD remains unidentified. Experiments recently showed that alterations in autophagic processes play a key role in the advancement of cardiovascular problems.^[3]^ It has been demonstrated that excessive autophagic activity promotes plaque destabilization and the death of smooth muscle cells in atherosclerosis.^[11,12]^ Furthermore, knocking down *Atg7* in smooth muscle cells in an experimental model reduces autophagic activity, which has negative consequences such as increased cellular senescence, neointima development, and severe atherosclerosis.^[13,8]^ Subsequently, defective autophagy by lacking *Atg7* and apolipoprotein E in mice has been reported to contribute to the development of plaque formation, instability, and rapture.^[13]^ Evidence is accumulating that autophagy induces eNOS expression, which further inhibits the production of inflammatory cytokines.^[14]^ In the present study, we assessed the association of ARG1 and nitric oxide metabolites in patients with CAD. Significantly high levels of serum ARG1 were found in patients than in healthy controls. Moreover, nitrite and nitrate were significantly lower in patient group than in the healthy controls. Evidence showed that overexpression of arginase-2 (ARG2) in endothelial cells reduces autophagy through the inhibition of the protein kinase, AMP-activated, α-catalytic subunit (PRKAA) signaling pathway.^[15]^ The findings were further confirmed by silencing of ARG2, which resulted in enhanced PRKAA and the formation of functional autophagosome.^[15]^ We studied the relationship between variant genotypes at *ATG7* and serum ARG1. Interestingly, we observed increased levels of ARG1 in CAD subjects having variant genotypes at *ATG7* rs1375206 (Chr3:11297643) and rs550744886 (Chr3:11272004). Albeit, no similar studies could be retrieved, but high concentration of ARG1 in the bloodstream of patients with cardiovascular disease has been reported earlier.^[16]^ The mechanism of high arginase resulting in altered autophagy in endothelial and in smooth muscle cells is not very clear. Xiong et al^[15]^ revealed that high ARG2 impaired endothelial autophagy. ARG1 and ARG2 share the common function of metabolism in vascular endothelial cells where both isoenzymes metabolize L-arginine to urea and ornithine, resulting in reduced levels of vasoprotective nitric oxide and endothelial dysfunction.^[17]^ Therefore, the findings of our high ARG1 levels and reduced nitric oxide metabolites in the carriers of variant genotypes at *ATG7* are in line with the earlier reports. However, more in-depth studies (in vivo and in vitro) are required to clarify the novel biological relation of arginase with autophagy as evidenced in the current study.

SNPs are not only useful markers for the identification of population divergence, but they can be utilized as candidate markers in complex diseases. Genetic variants in *ATG7* and their association with the disease are relatively novel, as limited data have been reported to show an association with human diseases. Gene encoding human ATG7 protein is located at chromosome 3p25.3,^[18]^ and several polymorphic sites have been identified and associated with the onset of Huntington disease,^[19]^ Breast cancer,^[20]^ and Parkinson disease.^[21]^ In the current study, we demonstrated an association between *ATG7* rs1375206 C/G and rs550744886 C/G polymorphisms and CAD in patients originating from Pakistan. In patients with CAD, the heterozygous CG genotype was substantially more common than in controls, followed by the variant GG homozygous genotype. The observed association in our study was justified and remains constant in multivariate logistic regression analysis. Our findings are consistent with earlier research that has found a link between *ATG7* polymorphisms and cardiovascular disease in patients.^[22]^ Concurrently, Zhang et al identified DNA sequence variants and SNPs in patients with acute myocardial infarction. In their functional analysis, *ATG7* rs550744886 (Chr3:11272004) G allele significantly altered the binding of transcriptional factors resulted in the reduced functional activity of *Atg7* promoter in the H9c2 cell line.^[3]^ At the population level, *ATG7* polymorphisms are yet to be clarified. There are no published data to show the allele frequency in CAD patients. Comparable to us, the variant allele at *ATG9B* rs2373929 increases the 1.7-fold high risk of CAD in patients than in controls.^[23]^ The polymorphic variant at *ATG7* rs1375206 (Chr3:11297643) was detected in Parkinson disease patients. The *ATG7* rs1375206 G allele was reported at 35.5% in patients, while it was 31.9% in healthy volunteers.^[21]^ The reported frequencies in previous literature are comparable with current findings of 41.3% G allele in CAD patients and 25.8% in healthy control subjects. The current study depicted the association of SNPs in *ATG7* with CAD in relation to arginase and nitric oxide metabolites. The baseline results provided here would be helpful in future studies to open the causal chain of ATG7 in CAD especially linked with the biochemistry of nitric oxide.

Despite enough sample size in the current study, results may be cautiously read as there are some limitations. The present study was limited to 2 SNPs in *ATG7*, and the protein level of ATG7 was lacking. Therefore, the variants in *ATG7* and their association with CAD must be read to the level of association with the disease; moreover, further controlled studies will be of great importance to evaluate the possible causal link between *ATG7* variants and CAD among the patients.

This study concluded a significant association of *ATG7* rs1375206 and rs550744886 polymorphism with CAD compared with the control group. Variant genotypes at *ATG7* rs1375206 and rs550744886 showed significant link with the altered serum levels of ARG1, nitrate, and nitrite among the patients with CAD.

The data used to support the current results are included in this article. For more information, please contact at sabirhussain@comsats.du.pk.

### Acknowledgments

The authors acknowledge all the volunteers and participants of the study and COMSATS University Islamabad for providing the facilities.

### Author contributions

Conceptualization: S.H., S.T.A.S., M.J.K., and S.M.N.;

data curation: M.S., S.F.A.S., S.M.N., and Z.M.;

formal analysis: M.S., S.F.A.S., and N.B.;

investigation: M.S. and S.F.A.S.;

methodology: M.S., S.F.A.S., and N.B.;

project administration: S.H. and S.M.N.;

resources: S.H., S.M.N., and S.T.A.S.;

software: S.F.A.S.;

supervision: S.H.;

validation: all authors;

writing—original draft: S.M.N., S.H., and S.T.A.S.;

writing—review and editing: M.J.K., Z.M., N.B., and S.H.
